# Preventing Nerve Function Impairment in Leprosy: Validation and Updating of a Prediction Rule

**DOI:** 10.1371/journal.pntd.0000283

**Published:** 2008-08-27

**Authors:** Ron P. Schuring, Jan H. Richardus, Ewout W. Steyerberg, David Pahan, William R. Faber, Linda Oskam

**Affiliations:** 1 KIT (Royal Tropical Institute) Biomedical Research, Amsterdam, The Netherlands; 2 Department of Public Health, Erasmus MC, University Medical Center Rotterdam, Rotterdam, The Netherlands; 3 Rural Health Program, The Leprosy Mission Bangladesh, Nilphamari, Bangladesh; 4 Department of Dermatology, Amsterdam Medical Centre, Amsterdam, The Netherlands; London School of Hygiene and Tropical Medicine, United Kingdom

## Abstract

**Background:**

To validate and update a prediction rule for estimating the risk of leprosy-related nerve function impairment (NFI).

**Methodology/Principal Findings:**

Prospective cohort using routinely collected data, in which we determined the discriminative ability of a previously published rule and an updated rule with a concordance statistic (*c*). Additional risk factors were analyzed with a Cox proportional hazards regression model. The population consisted of 1,037 leprosy patients newly diagnosed between 2002 and 2003 in the health care facilities of the Rural Health Program in Nilphamari and Rangpur districts in northwest Bangladesh. The primary outcome was the time until the start of treatment. An NFI event was defined as the decision to treat NFI with corticosteroids after diagnosis. NFI occurred in 115 patients (13%; 95% confidence interval 11%–16%). The original prediction rule had adequate discriminative ability (*c* = 0.79), but could be improved by substituting one predicting variable: ‘long-standing nerve function impairment at diagnosis’ by ‘anti-PGL-I antibodies’. The adjusted prediction rule was slightly better (*c* = 0.81) and identified more patients with NFI (80%) than the original prediction rule (72%).

**Conclusions/Significance:**

NFI can well be predicted by using the risk variables ‘leprosy classification’ and ‘anti-PGL-I antibodies’. The use of these two variables that do not include NFI offer the possibility of predicting NFI, even before it occurs for the first time. Surveillance beyond the treatment period can be targeted to those most likely to benefit from preventing permanent disabilities.

## Author Summary

Leprosy is caused by a bacterium that attacks the peripheral nerves. This may cause nerve function impairment (NFI), resulting in handicaps and disabilities. Therefore, prediction and prevention of NFI is extremely important in the management of leprosy. In 2000, a prediction rule for NFI was published, but circumstances have changed since the study was performed in the 1990s: the leprosy detection delay has shortened and the definition of NFI has changed. The original rule used ‘leprosy classification’ and ‘NFI present at diagnosis’ to predict future NFI. In the current patient population we studied an adjusted rule based on ‘leprosy classification’ and ‘presence of antibodies’. This adjusted rule predicted NFI more often than the original rule. With the adjusted rule it is now also possible to assess NFI risk before the first nerve damage event takes place. This may help doctors and health workers to improve surveillance for people at high risk. Early detection and treatment can then prevent permanent disabilities.

## Introduction

Preventing permanent disabilities due to nerve function impairment (NFI) [1] remains a major concern in leprosy control. *Mycobacterium leprae*, the causative agent of leprosy, infiltrates Schwann cells of peripheral nerve fibers [2]. Subsequently, the nerve fibers can be damaged by accumulation of bacteria and hypersensitivity reactions of the immune system. The decline of nerve function can take place before, during and/or after leprosy treatment. Early detection (within 6 months) and corticosteroid treatment may prevent further decline [3]. With leprosy control becoming less specialized and increasingly integrated into general health care services, there is a need for simplified procedures at the field level for timely identification and treatment of NFI in leprosy patients. The chances of preventing disability increase when health care workers pay special attention to patients who have a high risk of developing NFI.

To date, several risk factors for NFI have been identified [4]–[6], and an NFI prediction rule was formulated based on data from the Bangladesh acute nerve damage study (Bands) [4]. The Bands prediction rule categorizes patients into NFI risk groups based on their World Health Organization (WHO) classification (ie, paucibacillary [PB] or multibacillary [MB] leprosy) and the presence of long-standing NFI at diagnosis. However, validation of the Bands prediction rule is needed because i) the definition of NFI has since changed; ii) shorter detection delays have led to a smaller percentage of patients with NFI at diagnosis [7] which may change the contribution of this variable to the prediction rule; iii) a new and simple serological test for anti-phenolic glycolipid I (PGL-I) antibody detection [8],[9] has made routine screening feasible; and iv) no study has simultaneously assessed all known potential NFI risk factors, namely sex, age, WHO leprosy classification, long-standing NFI at diagnosis, bacterial load and anti-PGL-I antibodies [4]–[6].

We first validated the Bands NFI prediction rule. Next, we compared the performance of an adjusted NFI prediction rule, taking presence of anti-PGL-I antibodies into account.

## Methods

### Patients and procedures

Patients were previously untreated leprosy patients, newly diagnosed at the Rural Health Program (RHP) in northwest Bangladesh in 2002 and 2003. All patients participated in the Colep trial (ISRCTN 61223447) [10], which studied the effect of chemoprophylaxis in persons who had contact with leprosy patients (n patients = 1,037). Patients were classified as PB or MB according to the 1998 WHO classification [11] for treatment purposes. For the current analysis, patients who had experienced NFI for <6 months at the time of diagnosis (n = 162) were excluded as they required immediate treatment with corticosteroids, which influences the future occurrence of NFI, the primary outcome event of the study. Eleven patients were excluded because essential data were missing. This leaves a study population of 864 patients (538 males, 326 females; median age 34 years, range 5–84 years). Follow-up ended in September 2006 (median follow-up time 46 months). Patient information was prospectively recorded on standardised forms by the RHP staff.

The primary outcome was the time until the start of treatment. An NFI event was defined as the decision to treat NFI with corticosteroids after diagnosis. The decision was based on the guidelines described in the Rural Health Program (RHP, formerly DBLM) treatment protocol, [12] which states that a full dose course of prednisolone (starting with 40 mg/day and tapering off over 16 weeks for adults) should be given in case of i) nerve function reduction by ≥2 points in sensory and/or motor function tests of the ulnar, median, and/or posterior tibial nerves; ii) corneal anaesthesia; iii) a nerve tenderness score of 2; or iv) mixed mild symptoms of neuritis (ie, tenderness, sensory, and motor function scores of 1). The level of tenderness was defined as mild (score = 1) if palpation of the nerve causes some pain, but does not cause the patient to jump or cry out and defined as severe (score = 2) if touching the nerve causes the patient to jump or cry out. A low dose course of prednisolone (starting with 20 mg/day and tapering off over eight weeks for adults) is given for i) cutaneous neuritis; or ii) a mild skin reaction in a patch near or overlying a facial nerve. Thus, the criteria to treat NFI with prednisolone include all leprosy reactional and silent neuritis events. In both the Bands and the current study, sensory testing was performed with the Watson ball-point pen test, [13] motor function was assessed according to Medical Research Council grading [14], and changes in nerve function were evaluated by a physiotechnician trained in nerve function assessment.

Patients were under monthly surveillance during standard multidrug treatment (MDT): 6 months for PB patients, 12 months for MB patients. In the original Bands study [4] recommendations for extended surveillance were formulated, stating that for the low-risk group—PB patients without long-standing NFI at diagnosis—routinely performed surveillance for NFI during MDT is sufficient and health education should be provided so that patients are able to recognise and report NFI after completion of MDT. Medium-risk group patients—PB patients with and MB patients without long-standing NFI at diagnosis—require 12 months of surveillance and health education, implying that extended surveillance is only necessary for PB patients, who receive 6 months of MDT. For the high-risk group—MB patients with long-standing NFI at diagnosis—24 months of surveillance is recommended, resulting in 12 months of surveillance in addition to the routine follow-up during MB MDT.

The bacterial load was determined by microscopy on Ziehl-Neelsen stained slit skin smears [15] taken from the earlobe, forehead and a skin lesion. The bacterial load was positive if any bacteria were detected in one of the smears.

The presence of IgM antibodies against M. leprae was determined at diagnosis with a previously described enzyme-linked immunosorbent assay (ELISA), [16] using dried blood on filter paper. Briefly, the terminal trisaccharide of phenolic glycolipid I (PGL-I) linked to bovine serum albumin via a phenolic ring (NT-P-BSA, kindly provided by Prof. T. Fujiwara, University of Nara, Japan) was used as a semisynthetic analogue [17]. The titer of IgM antibodies against M. leprae was expressed as net optical density (OD): the absorbance of NT-P-BSA minus that of BSA-coated wells at 450 nm. The status “seropositive” was assigned if the net OD was ≥0.20.

### Ethical implications

This study uses data and samples that are routinely collected by the Rural Health Program from all leprosy patients before, during and after treatment and when patients undergo leprosy reactions. All patients included here gave written informed consent to participate in the Colep trial (ISRCTN 61223447) [10], a study approved by the Bangladesh Medical Research Council (BMRC/ERC/2001-2004/799). By giving written informed consent to participate in Colep and accepting treatment they agreed that their data could be used anonymously for research.

### Statistical analysis

Kaplan-Meier survival curves were used to determine the cumulative incidence of NFI for the risk groups defined by the prediction rules. Discriminative ability was expressed as a concordance (*c*) statistic [18] (range 0.5–1.0). Cox proportional hazards regression was used to identify independent variables that influenced the hazard ratio for NFI. The results are expressed as rate ratios or hazard ratios. Variables associated with NFI in univariate analyses (p<0.10) were selected for multivariable analysis in which stepwise backward selection was used to lessen the number of predictors, inclusion at p<0.05. Interactions between variables were tested but not included because they had limited predictive effects. The total number of monthly surveillances was calculated by multiplying the number in a risk group with the recommended surveillance period. The formula for routine surveillances was [(n PB*6)+(n MB*12)], and for surveillance based on the prediction rule the formula was (n low risk*6)+(n medium risk*12)+(n high risk*24)]. The number of surveillances needed to detect 1 case is the total number of surveillances/NFI cases found. Data analyses were performed with SPSS for Windows (version 14.0 SPSS Inc., Chicago, IL) and R software (version 2.3.1 www.r-project.org).

## Results

NFI occurred in 115 of 864 patients (13%; 95% confidence interval [CI] 11–16%).

**Figure 1:**
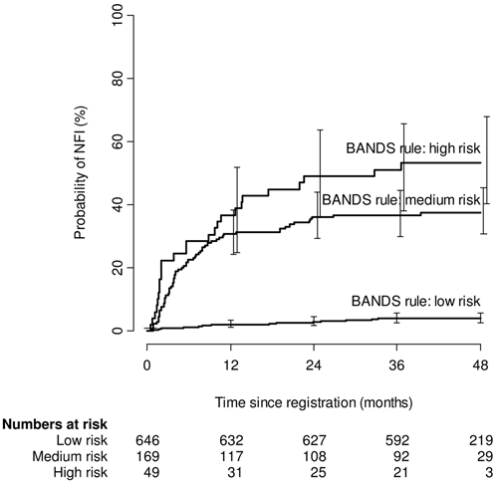
Cumulative incidence of NFI for risk groups defined by the Bands prediction rule, using WHO leprosy classification and longstanding NFI at diagnosis as predictive variables. NFI = nerve function impairment, Bands = Bangladesh acute nerve damage study.

The Bands prediction rule defines NFI risk groups according to the WHO leprosy classification (PB/MB) and longstanding NFI at diagnosis. The low-risk group, comprised of PB patients without longstanding NFI at diagnosis, had a cumulative NFI incidence of 4.0% (95% CI 2.8–5.9% [Figure 1]), the medium-risk group—PB patients with and MB patients without longstanding NFI at diagnosis—of 37% (95% CI 30–45%) and the high-risk group—MB patients with longstanding NFI at diagnosis—of 53% (95% CI 40–68%). The cumulative incidences of NFI between the medium- and high-risk groups did not differ significantly.

**Figure 2:**
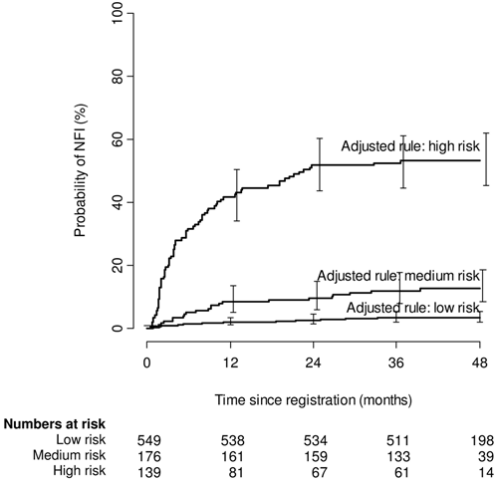
Cumulative incidence of NFI for risk groups defined by the adjusted prediction rule, using WHO leprosy classification and presence of anti-PGL-I antibodies as predictive variables. NFI = nerve function impairment, PGL-I = phenolic glycolipid I.

Substituting ‘long-standing NFI at diagnosis’ with ‘anti-PGL-I antibodies’ resulted in risk groups with cumulative incidences similar to those observed in the original Bands study (Figure 2) [4]. With the adjusted prediction rule the low-risk group—seronegative PB patients—had a cumulative incidence of NFI of 3.5% (95% CI 2.2–5.4%), the medium-risk group—seropositive PB patients and seronegative MB patients—of 13% (95% CI 8.5–19%), and the high-risk group—seropositive MB patients—of 53% (95% CI 45–62%). The cumulative incidences of NFI differed significantly between low-, medium-, and high-risk groups. The cumulative incidence of this medium-risk group is much lower than the 37% in the medium-risk group defined by the Bands prediction rule.

Statistical analyses (Table 1) evaluated the association of NFI with sex, age, WHO classification, long-standing NFI at diagnosis, bacterial load, and anti-PGL-I antibodies. All variables but age were univariately associated with NFI (p<0.05). A multivariable analysis indicated that ‘WHO classification’ and ‘anti-PGL-I antibodies’ were significantly associated with NFI (p<0.0001). MB patients were at an increased risk of NFI (HR 8.0, 95% CI 5.0–13.0) compared to PB patients, and seropositive patients had an increased hazard risk of 2.9 (95% CI 1.8–4.6) compared to seronegative patients. When adjusted for WHO classification, the variables sex, age, bacterial load, and longstanding NFI at diagnosis were not significantly associated with NFI anymore.

**Table 1:** Cox proportional hazards regression analysis, determination of NFI risk factors.

Bands rule	Adjusted rule			Total
	Low risk	Medium risk	High risk	
Low risk	531 (18)	115 (8)	0	646 (26) 4.0%
Medium risk	18 (1)	54 (13)	97 (49)	169 (63) 37.3%
High risk	0	7 (1)	42 (25)	49 (26) 53.1%
Total	549 (19) 3.5%	176 (22) 12.5%	139 (74) 53.2%	

a HR = hazard ratio.

b data missing for 14 patients.

CI = confidence interval, NFI = nerve function impairment.

The observed *c* statistic for the Bands prediction rule in our study was 0.79. The *c* statistic for the adjusted prediction rule was 0.81, showing a better discriminative ability. Table 2 shows the number of patients that would be classified differently with the adjusted prediction rule compared to the Bands prediction rule. The adjusted prediction rule would place 115 of the low-risk group patients in the medium-risk group and 97 of the medium-risk group patients in the high-risk group; only 18 patients from the medium-risk group and seven patients from the high-risk group would be placed in a lower risk group.

**Table 2:** Agreement between NFI risk groups according to the Bands prediction rule and the adjusted prediction rule.

Variables	Number	NFI event	Univariate		Multivariable (full model)		Multivariable (selected)	
			HR^a^	95% CI	HR^a^	95% CI	HR^a^	95% CI
**All patients**	864	115						
**Sex**								
Male	538	87	1		1			
Female	326	28	0.5	0.3–0.8	0.8	0.5–1.2		
**Age (years)**								
<15	136	11	0.6	0.3–1.2	0.7	0.3–1.3		
15–29	294	39	1		1			
30–39	161	25	1.2	0.7–2.0	1.1	0.6–1.8		
≥40	273	40	1.1	0.7–1.7	0.9	0.6–1.3		
**WHO leprosy classification**								
PB	669	29	1		1		1	
MB	195	86	13.4	8.8–21	7.5	4.4–13.0	8.0	5.0–13.0
**longstanding NFI at diagnosis**								
No	792	86	1		1			
Yes	72	29	4.4	2.9–6.8	1.3	0.9–2.1		
**Bacterial load** b								
Negative	759	66	1		1			
Positive	91	48	8.2	5.7–12.0	1.0	0.6–1.6		
**Anti-PGL-I serology**								
Negative	605	31	1		1		1	
Positive	259	84	7.5	5.0–11.3	2.7	1.6–4.5	2.9	1.8–4.6

Table shows number of patients per risk group and (number of patients with NFI event). Totals show number of patients, (number of patients with NFI event) and percentage of NFI events in that particular group.

NFI = nerve function impairment, Bands = Bangladesh acute nerve damage study.

Seventy-six (76/115, 66%) NFI events occurred while patients were undergoing routine surveillance. For the remaining 39 NFI events, additional surveillance would have been necessary for early detection. Extended surveillance using the Bands prediction rule [4] led to the detection of an additional seven patients with NFI for a total of 83 (83/115, 72%: 726 extra visits needed). Using the adjusted prediction rule, the number of additional detected patients with NFI increased to 16, for a total of 92 (92/115, 80%: 2388 extra visits needed). With routine surveillance, 83.6 visits led to the detection of 1 case, for the Bands prediction rule this was 85.3, and for the adjusted prediction rule 95.0.

## Discussion

Predicting NFI is important for identifying new leprosy patients that are at risk for nerve damage and, consequently, permanent disability. We describe an adjusted NFI prediction rule that replaces the variable ‘longstanding NFI at diagnosis’ with ‘anti-PGL-I antibodies’. The adjusted prediction rule was better able to identify patients at risk of developing NFI after diagnosis.

The original Bands prediction rule for NFI is based on WHO leprosy classification and long-standing NFI at diagnosis [4]. A Kaplan-Meier survival analysis showed that the medium- and high-risk groups had similar survival curves (Figure 1), indicating that the Bands prediction rule could not differentiate between these two groups. One explanation may be that the definition of NFI has changed since the Bands study: a new NFI category, with less serious events that require a low dose course of prednisolone, was added to original NFI events that require a full dose course [12]. This leads to more patients being identified at an early stage of NFI. In addition, a smaller percentage of long-standing NFI (>6 months) and a higher percentage of recent NFI (<6 months), due to shorter detection delays, may have changed the contribution of longstanding NFI at diagnosis.

Presence of anti-PGL-I antibodies against *M. leprae* are a well-known risk factor for NFI [5]. In-depth analysis of all known risk factors for NFI in the current patient cohort showed that NFI is best predicted by ‘WHO classification’ and ‘anti-PGL-I antibodies’ (Table 1). We adjusted the Bands prediction rule by replacing ‘long-standing NFI at diagnosis’ by ‘anti-PGL-I antibodies’. The adjusted rule was able to differentiate between three risk groups with significantly different cumulative incidences of NFI (Figure 2); the *c* statistic increased from 0.79 to 0.81. Unfortunately, we could not validate the adjusted prediction rule on the original Bands cohort because no serology data were available.

The adjusted prediction rule distinguished three risk groups comparable to those in the Bands study [4] (Figure 2). Therefore, the surveillance recommendations that were based on the Bands study [4] can be maintained (see Methods). When replacing the Bands prediction rule with the adjusted prediction rule 212 patients were reassigned to a higher risk group and 25 patients to a lower risk group (Table 2), suggesting that the adjusted prediction rule has considerable implications for patient care. The reassignment of these patients to a higher risk group is warranted because they have a higher-than-average risk to develop NFI: 7% for patients moving from the low to the medium risk group and 51% for patients moving from the medium to the high risk group. The adjusted prediction rule can thus be used to identify a substantially higher number of new NFI cases than either routine or Bands rule-based surveillance and offers increased opportunity to prevent nerve damage in leprosy. However, the number of visits needed to detect one case is higher than with alternative strategies. We consider this operationally feasible and medically justifiable in view of the serious consequences of NFI, including life-long disability.

WHO classification is a good predictor of future NFI [6] but it rather crudely divides leprosy patients into two groups (PB and MB). The presence of anti-PGL-I antibodies is known to correlate with the bacterial load [16], and thus offers a further refinement of the WHO classification into patients with high and low bacterial loads. This may explain the added predictive value of the presence of antibodies. In contrast to the Bands rule the adjusted rule uses two variables that do not include NFI. This offers the possibility of predicting NFI before it actually occurs.

We expect that the adjusted NFI prediction rule will be relevant in other settings, since the predicting variables are well defined and easily determined, but it should be validated externally. We believe that the adjusted prediction rule can be applied in current health services, since it fulfils the need for simplified guidelines and diagnostic protocols. Contrary to the Bands prediction rule, the adjusted rule does not rely on a specialist physiotechnician for the prediction. However, this person is needed to document the baseline nerve status and for surveillance during follow up examinations. Recently, a simple anti-PGL-I field test was developed that gives results within ten minutes, [8],[9] making routine testing feasible. Thus, leprosy diagnosis and NFI prediction can be accomplished during a single consultation. Additional benefits of the anti-PGL-I test are that it assists with the classification and aids diagnosis of leprosy patients with doubtful clinical signs [8],[9],[16].

With the adjusted prediction rule, the necessity to continue surveillance beyond the treatment period can be determined. New leprosy patients can be assigned to an NFI risk group, and appropriate surveillance can be planned. Nerve damage can thus be successfully prevented despite the fact that leprosy control has been integrated into general health services.
